# The Effect of Girth Design and Girth Tension on Saddle-Horse Pressures and Forelimb Stride Kinematics in Rising Trot

**DOI:** 10.3390/ani15172540

**Published:** 2025-08-29

**Authors:** David Marlin, Olivia Randell, Emma Mayhew, Roberta Blake

**Affiliations:** 1 Animalweb Ltd., The Granary, Hermitage Court, Hermitage Lane, Maidstone ME16 9NT, UK; 2 Agriculture, Animal and Environmental Sciences, Anglia Ruskin University, Lordship Rd, Writtle, Chelmsford CM1 3RR, UK; olivia.randell@btinternet.com (O.R.); ejmvetphysio@gmail.com (E.M.); roberta.blake@aru.ac.uk (R.B.)

**Keywords:** riding, exercise, equestrian, biomechanics, tack

## Abstract

The aim of this study was to investigate the effect of girth design and girth tension; six horses regularly ridden were used. Each horse underwent four experimental sessions in an unbalanced Latin-square design with two girth tensions (8 kg or 16 kg) and two girth designs (straight girth (S) or anatomical girth (A)). Pressure between the saddle and the horse was measured at 100 Hz with a pressure mat (0.5 sensels per cm^2^). Notably, 2D limb kinematics were determined from anatomical markers placed on the fore and hindlimbs. Video was collected at 240 fps. There was no significant effect of girth type, girth tension, or girth type*tension interaction for any of the measured variables, with the exception of carpal flexion, which was significantly greater for A8 (median: 103°, 25th–75th percentile: 100–112°) than S8 (101°, 96–106°; *p* = 0.043). There was no effect of girth type (A or S) on mean saddle pressure for either cranial or caudal regions (*p* > 0.05), but caudal average pressure was significantly lower than cranial average pressure both at 8 and 16 kg tensions (*p* < 0.05). For both mean and peak pressure, the ratio cranial: caudal was significantly higher with 16 kg tension (*p* < 0.05), indicating that as the girth tension increases, the pressures shift towards the cranial aspect. In conclusion, neither girth tension nor girth type significantly influenced 2D limb kinematics, but higher tension has shifted the load towards the cranial area significantly, which could contribute to cranial thoracic back pain or injuries.

## Simple Summary

Horse tack, including the girth (the strap that holds the saddle in place), can influence how comfortable and free-moving a horse feels when being ridden. This study explored how different types of girths and the tightness of the girth (tension) affect pressure beneath the saddle and the movement of the horse’s forelimbs during a rising trot. Six regularly ridden horses were tested using two types of girths—a straight design and an anatomical (shaped) design—at two levels of tightness. We found that changing the girth type or tightness had very little effect on how the horse’s limbs moved. However, increasing the tightness of the girth caused a noticeable shift in saddle pressure towards the front part of the horse’s back. This pressure shift might be important because it could contribute to discomfort or back problems over time. Our findings suggest that while tightness and girth shape may not immediately change how a horse moves, higher girth tension can affect saddle fit and pressure distribution, which might impact horse comfort and performance in the long term.

## 1. Introduction

The girth, or cinch in Western riding, is a strap used to secure the saddle in place on ridden horses. A large variety of materials is used in girths, including leather, elastic, nylon, neoprene, cotton, wool, and PVC, and girths also come in a large range of designs, including straight, anatomical (shaped or contoured), string, dressage, jumping short, and full-length. Girths usually have two buckles that attach to the billets (girth straps) fixed to the saddle, of which there are commonly either two or three. Whatever the configuration, combined with the saddle, the girth forms a relatively inflexible ring around the horse at the level of T7 to T10. The girth overlays both the *m.serratus ventralis thoracis* and the *m.pectoral ascendens* whilst the saddle is pulled down onto the *m.spinalis dorsi* and the *m.longissimus dorsi.* These muscles play pivotal roles in both posture and locomotion and girth tensions required to provide saddle stability; therefore, they have the potential to impact stride kinematics. Thus, whilst there has been interest in and speculation about the extent to which restriction by the saddle-girth system may potentially negatively impact both ventilation and locomotion, interestingly, there have been relatively few published studies. The recent survey by the FEI Equine Ethics and Wellbeing Commission has raised concerns around tack, and there has been evidence that the stakeholders would like to see more research, with tack being underpinned as one of the priority areas [1].

In racehorses, average resting girth tensions of 9–13 kg have been reported, although values as low as 3 kg and as high as 22 kg have been recorded [2]. However, during the process of girth tightening, values as high as 53 kg were recorded, with 40 kg being an average peak value [2]. Bowers and Slocombe [3] investigated the effects of resting canvas girth tensions of 5, 10, 15, and 20 kg on peak girth tensions in a combination of thoroughbred and standardbred horses during galloping on a treadmill. Peak expiratory tension was similar to that set at rest, but mean peak tension at inhalation was 12.8, 22.3, 32.9, and 38.0 kg for 5, 10, 15, and 20 kg resting tension, respectively. Furthermore, run time to fatigue was significantly decreased at 10, 15, and 20 kg compared with 5 kg resting tension [3]. The same authors investigated the impact of girth material in a later study [4] and found that at initial tensions of 6, 12, and 18 kg, girths with elastic reduced peak inspiratory girth tension compared with canvas girths.

The reason why high girth tensions may limit performance has been subject to much speculation, with restriction of ventilation being frequently cited [3,4]. Marlin et al. [5] demonstrated that during canter and gallop exercise, horses exhibit paradoxical chest movements such that whilst abdominal circumference increases during inhalation, there is a small decrease in chest circumference at the level of the seventh/eighth ribs. In addition, simultaneous measurements of airflow and girth tension during canter show that when breath holding occurs, girth tension changes continue in phase with stride and are even elevated (Marlin, unpublished observation). Additionally, it has been shown that a 15 kg girth tension causes measurable changes in respiratory mechanics (loss of rib motion and increased pulmonary resistance) [6]. Horses do not use chest expansion during canter and gallop to achieve ventilation and the tidal volume is driven by diaphragmatic movements. The likely explanation for the lack of chest expansion and contraction is that the forelimb and chest muscles effectively “lock” the frontal thorax to stabilize the shoulder in the absence of any bony attachment to the axial skeleton [7]. In a treadmill study on Thoroughbred horses run at 110%VO_2max_, Bowers et al. concluded that “increasing girth tension was not associated with changes in respiratory mechanical or gas exchange properties” [8].

Girth tension was measured at rest and during lunging exercise at initial resting tensions of 6, 10, 14, and 18 kg [9]. Girth tension increased from stand to walk to trot to canter and some differences were seen due to rein (i.e., left versus right). However, there are some methodological concerns with this study, as although the load cell was able to generate readings at 200 Hz, the signal was only sampled once per second, and thus some detail of the signal will have been lost.

The pressure and force under a range of “standard” girths and an anatomical girth design were investigated using a pressure mat under the girth in ridden horses [10]. Maximum force on the left side of the horse under the girth for the standard and anatomical girth was 344 N and 281 N, respectively, and for the right side was 328 N and 288 N, respectively, and these were significantly different. Girth placement also has a significant effect on saddle pressures, with traditional placement seeming equally good if not better than the V-system, and should thus be considered in saddle fitting [11]. It is also important to recognize that the size of the girth (length and width) needs to be suitable for each individual horse and that the shape of the horse’s thorax influences where the girth sits, how pressure is distributed, and therefore how it might influence the gait [12].

To our knowledge, this is the first study to understand the impact of girth tension on saddle pressure, an important welfare parameter during ridden work, and its associated effects on forelimb kinematics. The aim of the present study was to investigate the interaction between girth tension and girth type using a standard straight material girth and a leather anatomical girth at initial tensions of 8 and 16 kg.

## 2. Materials and Methods

### 2.1. Ethical Approval

Ethical approval was provided by the Agriculture, Animal, and Environmental Sciences Research Ethics Panel, reference number 1717. Informed owner consent was obtained, and each rider completed a physical activity readiness questionnaire (PAR-Q, short version) prior to the commencement of data collection.

### 2.2. Sample Population

A convenience sample of six horses, determined by the research equation approach [13], was used (Table 1), consisting of five geldings and one mare, with the mean ± standard deviation (SD) height of 162 ± 2 cm and a mean age of 10 ± 4 years. All horses were in regular ridden schooling work. Four of the horses were owned by private riders, and two were from the Anglia Ruskin University Equine Development Centre. All horses were ridden by their owner, with the exception of the two riding school horses, ridden by a student. Horses were ridden by the same rider across the four interventions to ensure consistency. All horses had their saddles checked and fitted a maximum of 3 months prior to the trial, but a basic visual check of the saddles was performed by the researchers. All horses were familiar with the environment. An arena habituation period was carried out for all horses prior to data collection.

### 2.3. Girth Tension and Girth Types

**Figure 1:**
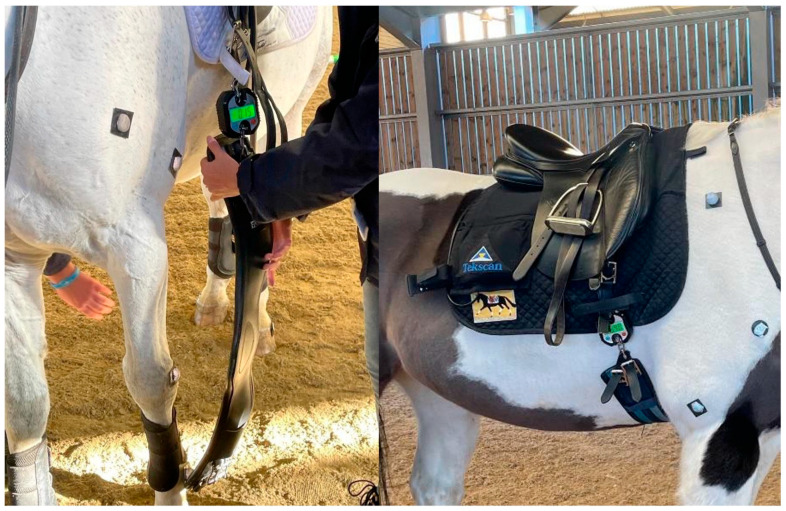
Tension gauge.

**Figure 2:**
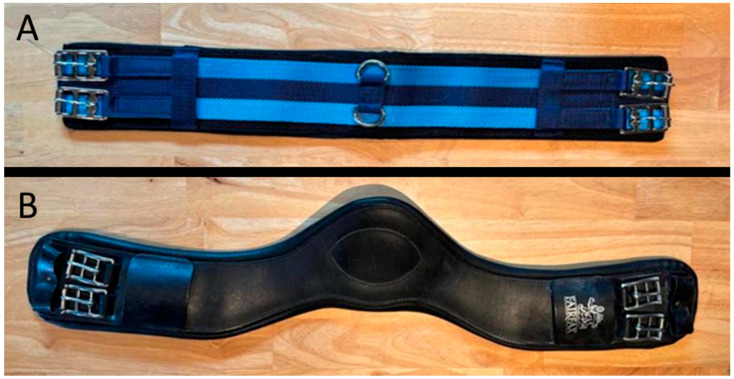
(**A**) Standard straight material girth (S) and (**B**) anatomical leather girth (F).

Initial (resting, pre-exercise) girth tension was set at 8 kg or 16 kg using an in-line load-cell gauge (SF-916, Jiangyin Suofei Electronic Technology Co., Ltd., Jiangyin, China) (Figure 1). The tensions were defined based on tensions identified or used in previous studies, which ranged from 6 kg to 20 kg [2,3,4,6,9]. Two girths were used (Figure 2): a standard straight girth (Unbranded) and an anatomical leather girth (A; Fairfax Short Dressage girth, Fairfax Saddles Ltd., The Saddlery, Walsall, West Midlands, WS3 2XJ, UK). All horses were ridden in their own bridles and saddles to mitigate the effects of ill-fitting tack [14]. All horses were ridden in general-purpose riding saddles, except for horse 2 which was ridden in a dressage saddle.

### 2.4. Kinetics Measurements

**Figure 3:**
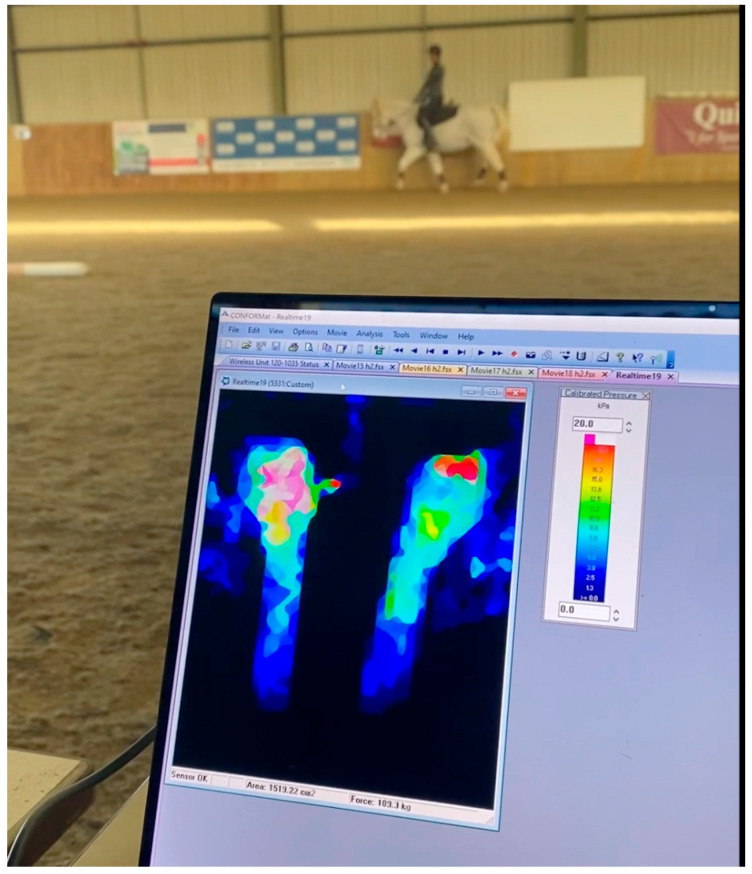
Example of pressure mapping from saddle pressures obtained during the experiment.

Pressure measurement between the saddle and horse was made with the Tekscan CONFORMat Tekscan system (Tekscan, Inc., 333 Providence Highway, Norwood, MA 02062, USA) which consists of a matrix of 32 × 32 individual resistive sensing elements (1024 in total) in a mat 47 × 47 cm giving a density of 0.5 sensels per cm^2^. Data were sampled at 100 Hz. Calibration was undertaken with the horse standing still, with the rider mounted and feet out of the stirrups. Data was collected and analyzed using the proprietary software CONFORMAT Research v7.6 (Tekscan, Inc. 333 Providence Highway, Norwood, MA 02062, USA). An example of data yielded can be seen in Figure 3.

### 2.5. Limb Kinematics

**Figure 4:**
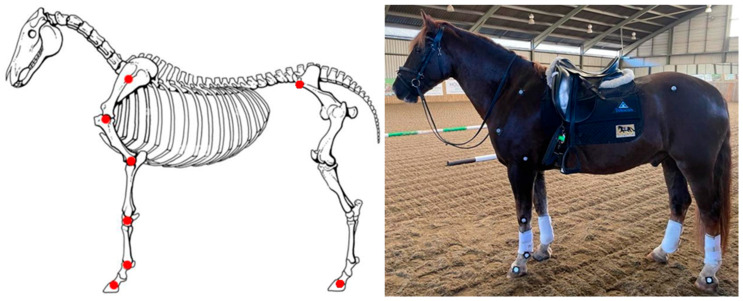
Location of anatomical markers.

Hemispherical reflective markers (20 mm diameter) were attached to both forelimbs using double-sided sticky tape as follows: the spine of the scapula; caudal humeral tuberosity; above the head of the radius; ulnar carpal bone; fetlock; coronet band (front and hind limbs); and tuber coxae (Figure 3). Palpation of these anatomical locations and placement of the markers were undertaken by the same author (OR) throughout the trial to improve consistency. Markers were applied before warm-up so the horses could habituate to them. All horses were ridden in their normal saddles so there would be minimal external influences on kinematics. Video calibration was performed at the beginning of each day by filming a meter-long rule in the center of the data collection channel (Figure 4). A camera (iPhone 15, Apple, Cupertino, California, United States) was used to capture 240 frames per second. The camera was set up on a tripod approximately 7 m perpendicular to the center of the marked data collection channel, with a second tripod holding a halogen light, which was used to illuminate the markers, placed directly behind it.

The slow-motion videos were uploaded onto a computer where 2D motion analysis software was used to analyze the videos (Quintic Biomechanics v35, Quintic Consultancy Ltd., Coleshill, UK). Software calibration was achieved using the video containing the 1-meter rule. Angles for the shoulder, elbow, carpus, and fetlock were analyzed throughout a full stride length, depicting the initial toe down-stance phase for the landing forelimb, toe off, and then the full swing phase to toe down again. All joint angles, protraction, and retraction were analyzed according to the relevant marker placements, and stride length was measured from toe down of one stride to toe down of the next. The maximum flexion and maximum extension values were recorded for each intervention. All whole strides within the 10 s video were analyzed.

### 2.6. Experimental Design and Protocol

The study utilized a within-subjects repeated measures design. The intervention order was semi-randomized (Table 2), to attempt to reduce order of treatment effect. This ensured that any order-related effects were distributed evenly across treatments, reduced the risk of systematic bias, and strengthened the validity of the results, allowing differences in pressure and biomechanics to be attributed more confidently to girth type and tension rather than external or confounding factors. Each horse received the four interventions within a session, with a 15-minute interval between interventions for rest, and new girth/girth tension was applied. For each horse, the horse was brought to the arena and the anatomical markers were positioned. The pressure mat was placed on the horse by the same author (EM) to try to ensure consistency. No saddle pad was used, besides the pad containing the pressure-mapping system. The saddle was placed on the horse and the girth was tightened with the tension gauge in-line (Figure 1). Once the rider had mounted, a few minutes were given to allow for stretching and compression of the mat, saddle, and girth. The girth was then loosened and the mat was calibrated to the weight of the rider and saddle. Prior to data collection, each horse underwent its usual warm-up routine for approximately five minutes to allow for acclimatization to the arena. The warm-up and data collection were carried out in the same arena (60 × 24 m) on an Andrews Bowen Propell surface. All data collection took place over 2 days. Once warmed up, the required girth tension for the first intervention was then achieved by tightening at the end of exhalation to either 8 kg or 16 kg. Horses completed three passes through the alleyway, at rising trot, on each rein, in which ten seconds of data were collected. Passes were discarded if the horses changed gait or showed any inconsistency in gait. In between interventions, the girth was completely loosened, a rest break was given, and the new tension or new girth was applied.

### 2.7. Data Analysis and Statistical Analysis

Difference score (%) cranial:caudal was calculated by the equation:

Difference score (%) = (cranial − caudal)/caudal

The cranial area of the saddle is considered along with the cranial half of the contact area of panels and caudally the caudal half of the contact area of panels.

Statistical analysis was undertaken using SPSS software (v29.0, IBM, Armonk, New York, NY, United States).

#### 2.7.1. Kinetics Data

Shapiro–Wilk test indicated that all kinetics data assumed a normal distribution (*p* > 0.05); therefore, a two-way repeated measures ANOVA was used to determine main effects of girth (A or S) and tension (8 kg or 16 kg) and interaction effects of girth*tension. Data are presented as mean ± SD with significance set at *p* < 0.05.

#### 2.7.2. Kinematics Data

Shapiro–Wilk test indicated that for limb kinematics, data were parametric for all data, except for retraction. Data with normal distribution (*p* > 0.05 on Shapiro–Wilk test) were analyzed with a two-way repeated measures ANOVA to determine main effects of girth (A or S) and tension (8 kg or 16 kg) and interaction effects of girth*tension. Non-parametric data (*p* < 0.05 on Shapiro–Wilk test) and Wilcoxon’s rank tests (for non-parametric pairs) or paired *t*-tests (for parametric pairs) were used to analyze the data.

## 3. Results

All horses completed all interventions. Data for the left and right rein passes were combined.

### 3.1. Kinematics

Results for all kinematic variables are available in Supplementary Table S1.

There was no significant effect of girth type, girth tension, or girth type*tension interaction for any of the measured variables, except for carpal flexion.

**Figure 5:**
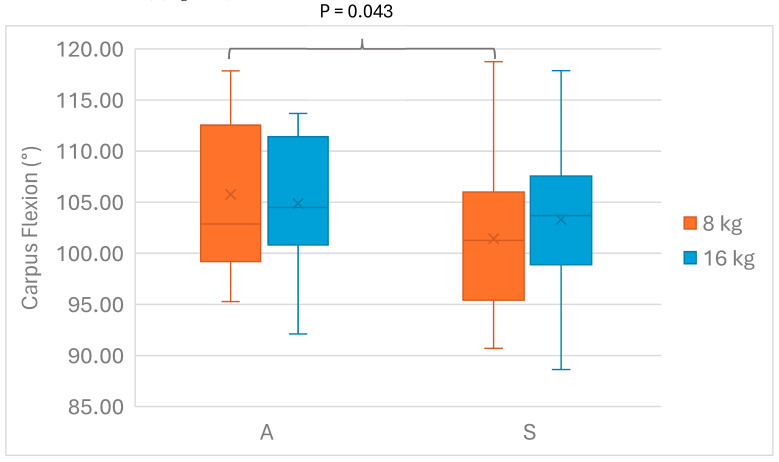
Carpal flexion angle (°) for anatomical (A) and straight (S) girths with 8 kg and 16 kg of tension. Median, 25th and 75th percentiles, minimum and maximum. The horizontal line represents the median, and “x” represents the mean. *n* = 6 horses.

When we investigated the main and interaction effects of girth design and tension on forelimb carpus flexion, we found that the interaction effect Design*Tension on carpus flexion was not significant (F(1,5) = 2.824, *p* = 0.154). Furthermore, there was no significant main effect for tension on carpus flexion (F(1,5) = 0.068, *p* = 0.804); however, there was a significant main effect for design on carpus flexion (F(1,5) = 7.317, *p* = 0.043). Post hoc analysis with a Bonferroni adjustment revealed that carpal flexion was statistically significantly higher with A8 when compared to S8 (−2.954 (95% CI, −5.760 to −0.147)°, *p* = 0.043) (Figure 5).

### 3.2. Kinetics

Results for the kinetics variables are available in Supplementary Table S2.

There was no effect of girth type on mean saddle pressure for either cranial or caudal regions, but the 16 kg tension increased the mean saddle pressure on the cranial area of the saddle, in comparison with the 8 kg tension (F(1,5) = 10.81, *p* = 0.022).

**Figure 6:**
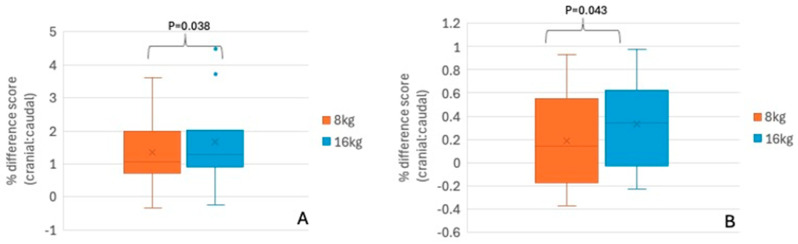
Mean (**A**) and peak (**B**) saddle pressure difference score cranial: caudal (%) for all girths combined with 8 kg and 16 kg of tension. Median, 25th and 75th percentiles, minimum and maximum. Horizontal line represents median and “x” represents mean. *n* = 6 horses. Cranial area of the saddle = cranial half of the contact area of panels; caudal area of the saddle = caudal half of the contact area of panels.

When analyzing the % difference score between the caudal and cranial areas of the saddle mean pressure distribution, there was a main effect of girth tension, F(1,5) = 7.832, *p* = 0.038, with a considerably higher loaded cranial area with the 16 kg tension in comparison with the 8 kg tension, a mean difference of 32.9%. This means that as the tension increased to 16 kg, the mean pressure significantly moved towards the cranial area of the saddle (Figure 6A).

Peak saddle pressure was not significantly different between regions or girth type or girth tension. However, when analyzing the % difference score between cranial and caudal areas of the saddle, peak pressure showed a significant shift towards the cranial area in the 16 kg tension, with 33.2% more peak pressure, on average, on the cranial area with 16 kg tension in comparison with 8 kg tension (F(1,5) = 7.310, *p* = 0.043) (Figure 6B).

For the mean force (N) under the saddle, there was no main effect of girth type for either cranial or caudal regions, but the 16 kg tension significantly increased the mean force on the cranial area of the saddle, in comparison with the 8 kg tension (F(1,5) = 9.43, *p* = 0.028).

## 4. Discussion

Mean pressures in the present study are similar to those previously reported for rising trot [15,16]. A ratio of ~2.5 to 1 for cranial to caudal mean saddle pressure for a correctly fitted saddle has also been reported previously [16], as seen in the present study.

As far as we are aware, this is the first study to investigate the effect of different girth tensions on saddle pressures. Neither average nor peak saddle pressure was affected by girth tension, despite quite a large difference (8 kg versus 16 kg) and the fact that we had anticipated that an increase in girth tension would result in an increase in pressure between the saddle and horse. However, we have observed a significant shift in the mean and peak pressures towards the cranial part of the saddle as the tension increased. The rationale for expecting an increase is that the saddles and girths used effectively produce a largely inflexible “ring” around the horses’ frontal thorax. The pressure concentration over the cranial aspect of the saddle has led to a less homogeneous pressure distribution under the panels, potentially decreasing local flexion-extension range of motion [17] which can have an impact on welfare and performance. It is established that girth tension causes loss of rib motion and increased pulmonary resistance in standing horses [6], and during movement, the tension shows cyclical changes related to both breathing and/or stride in horses. Chest movements at the level of the seventh rib, which is the approximate location of where the girth sits, at rest and during trot in unridden horses exercising on a treadmill, have been shown to correlate with ventilation, whilst at canter and gallop, they are unrelated to ventilation and are related to forelimb position and muscle volume underlying the girth. There may be several explanations as to why we did not see an impact of girth tension on overall saddle pressure. The first is that the riders’ contribution to saddle pressure, given a mass of around 75 kg and a force of 150 kg at 1.5 g acceleration in rising trot, far exceeds the force contribution of the girth. In addition, whilst the girth tension was set prior to exercise and confirmed after exercise, as tension was not measured during the test, it is conceivable that the compensation by the horse may have reduced the impact of the different pre-exercise tensions.

Wright [9] investigated the effect of pre-exercise girth tensions of 6, 10, 14, and 18 kg in Hanoverian horses lunged on a circle. The girth used was an inflexible (in terms of length) padded material girth. As data were only collected at 1 Hz, peak values are unreliable, but the average reported values may be acceptable. At a walk there was minimal change in mean girth tension from that at rest, but at a trot and canter the 6 kg resting tension had increased to a mean of 7.1 (+18%) and 9.6 kg (+60%), respectively, whilst for the 18 kg resting tension, the increase at a trot was to a mean of 21.3 kg (+18%) and 24.3 kg (+35%) at a canter. Given that in galloping horses, initial tensions of only 10 kg result in a reduction of run time to fatigue of 14% compared with an initial tension of 5 kg, it would be surprising if girth tensions of this level did not have some effect on horses’ performance.

Bowers and Slocombe compared elasticated and canvas girths but did not study leather girths [4,8]. In the present study, both girths, although constructed of different materials (leather and material), can be considered to be relatively inelastic, which may have accounted for the lack of influence of girth design/material on the saddle pressures seen with elastic materials [8]. Furthermore, although it is known that the saddle pressures change with girth strap placement [11], in our case, the placement of the strap was the same, which can further explain the absence of influence of design on the saddle pressures. Murray et al. [10] investigated pressure beneath a variety of unspecified girths and an anatomically shaped girth and reported that the anatomical girth reduced peak under-girth pressures and improved limb protraction and carpal/tarsal flexion in rising trot. Whilst Murray et al. found significant differences in girth design, specifically that the anatomical girth performed better, it is perhaps surprising that in the present study, we found a significant effect of tension but not girth type/material. One important difference is that Murray et al. used elite horses and riders [10] whilst in the present study, the horses and riders were not elite. It is also conceivable that standardization of girth tension negated any difference between the girth types.

In the study of Murray et al. [10], girth tension was not measured. Instead, the authors reported that “the girth was tightened symmetrically to the tension that the rider normally used”. Whilst the tensions commonly used in racing have been reported [2], we are not aware of any studies investigating tensions commonly used in other equestrian disciplines. Resting girth tension in the study of Bowers and Slocombe [2] ranged from 3 to 20 kg after tightening but before exercise. This was used as the basis to set the two tensions used in the present study.

Given that girth design, girth material, and girth tension have all been shown to influence limb kinematics and onset of fatigue [3,10], it is surprising that this area has received so little attention, and clearly there is scope for further research examining the interaction of girth material, girth design, girth tension, and gait. Research should also be undertaken to describe the typical pre-exercise girth tensions used by riders competing in dressage, jumping, and cross-country.

While the present findings contribute to understanding how girth tension influences equine biomechanics and saddle pressures, further research is warranted to clarify the underlying mechanisms and broader implications. One aspect that could be explored is different gaits like walking, sitting trot, cantering, and jumping. Future studies should investigate the effects of girth tensions on back and hindlimb kinematics, which can be affected by high back pressures, as it is well known that biomechanically, the head, neck, back, and hindquarters are connected and move together. A further area would be making correlations between both locomotor patterns and physiological responses. Longitudinal studies incorporating different horse breeds, ages, disciplines, and levels of training would help establish whether the observed biomechanical changes are consistent across populations. Additionally, integrating muscle activity (sEMG or AMG) analyses may provide deeper insights into the interaction between girth tension and musculoskeletal function. Exploring the relationship between girth tension and indicators of welfare, such as stress markers or behavioral responses, could also inform best practices for optimizing performance while safeguarding equine wellbeing.

The main limitation of this study is the small number of horses. For example, to detect a change of 5 kPa from a mean of 15 ± 5 kPa in the present study, it only has the power of 46%.

## 5. Conclusions

In conclusion, girth tension and girth type had minor effects on limb kinematics, and regarding kinetics, the main effect seemed to be a significant shift in the mean pressure and peak pressure towards the cranial aspect of the saddle in non-elite horses and riders in rising trot.

## Figures and Tables

**Table 1:** Sample population.

Horse	Age (years)	Gender	Breed	Height (cm)
1	13	Gelding	Connemara	163
2	9	Mare	Dutch Warmblood	163
3	10	Gelding	Irish Sports Horse	163
4	4	Gelding	Irish Sports Horse	160
5	16	Gelding	Irish Sports Horse	163
6	10	Gelding	Irish Cob	158

**Table 2:** Order of intervention for girth type (anatomical—A or standard—S) and tension (8 = 8 kg or 16 = 16 kg) for each subject.

Horse	Intervention 1	Intervention 2	Intervention 3	Intervention 4
1	A8	A16	S16	S8
2	A16	A8	S8	S16
3	S16	S8	A8	A16
4	A8	A16	S16	S8
5	S16	S8	A8	A16
6	A8	A16	S16	S8

## Data Availability

The dataset is available on request from the authors.

## Supplementary Materials

The following supporting information can be downloaded at: https://www.mdpi.com/article/10.3390/ani15172540/s1, Table S1: Mean ± standard deviation and Median (25th–75th percentile) for stride length (m), forelimb protraction, hindlimb protraction, shoulder, elbow, carpus and fetlock flexion, extension and ROM angles (°) for standard (S) and anatomical girths (A) at 8 and 16 kg tension (*n* = 6 horses). Table S2: Mean ± standard deviation for Mean Pressure (kPa), Peak Pressure (kPa) and Mean Force (N), on the cranial and caudal areas of the saddle, for standard (S) and anatomical girths (A) at 8 and 16 kg tension (*n* = 6 horses).

## Abbreviations

The following abbreviations are used in this manuscript:

ROM	Range of Motion
A	Anatomical Girth
S	Straight Girth
