# Supplementary material for: The Effect of Girth Design and Girth Tension on Saddle-Horse Pressures and Forelimb Stride Kinematics in Rising Trot
Source: Animals (Basel). 2025 Aug 29;15(17):2540. doi: 10.3390/ani15172540 (PMC12427339; doi:10.3390/ani15172540)
Supplement: Supplementary file 1 [file animals-15-02540-s001.zip › animals-3742936-supplementary.pdf]

Table S1. Mean ± standard deviation and Median (25th-75th percentile) for stride length (m), forelimb protraction, hindlimb protraction, shoulder, elbow, carpus and fetlock flexion, extension and ROM angles (°) for standard (S) and anatomical girths (A) at 8 and 16kg tension (n=6 horses).

	S8	S16	A8	A16	Girth Type (S v A)	Girth Tension (8 v 16kg)	Girth Type*Tension
Stride length (m)	3.07±0.69<br>2.98 (2.44-3.60)	3.14±0.69<br>3.25 (2.48-3.87)	3.04±0.69<br>2.97 (2.46-3.46)	3.13±0.70<br>2.94 (2.54-3.84)	F(1,5)=0.602, P=0.473	F(1,5)=0.953, P=0.374	F(1,5)=0.071, P=0.800
Forelimb protraction (°)	20.30±1.62<br>20 (20-21)	20.18±1.31<br>20 (19-21)	19.54±1.71<br>19 (18-21)	20.42±1.66<br>20 (19-22)	F(1,5)=0.747, P=0.427	F(1,5)=1.252, P=0.314	F(1,5)=3.072, P=0.140
Forelimb retraction (°)	19.06±2.31<br>19 (17-19)	19.23 ± 2.28<br>19 (18-21)	20.26 ± 1.70<br>20 (19-22)	19.33 ± 1.50<br>19 (19-20)	S8 vs A8= z = -1.782, p=0.075<br>S16 vs A16= t(5)=0.144, P=0.891	S8 vs S16= z = 0.314, p=0.753<br>A8 vs A16= t(5)=2.231, P=0.076	NA
Shoulder							
Flexion (°)	94.98±6.54<br>93 (90-98)	97.61±5.40<br>98 (94-103)	96.89±6.27<br>98 (92-101)	97.90±6.09<br>98 (94-103)	F(1,5)=0.014, P=0.911	F(1,5)=0.469, P=0.524)	F(1,5)=0.008, P=0.933
Extension (°)	106.32±9.16<br>104 (100-109)	107.83±6.48<br>106 (104-113)	106.69±7.79<br>106 (102-109)	107.91±7.65<br>106 (104-116)	F(1,5)=0.014, P=0.911	F(1,5)=0.469, P=0.524	F(1,5)=0.008, P=0.933
ROM (°)	11.34±7.24<br>9 (7-12)	10.21±2.52<br>10 (8-11)	9.80±2.90<br>9 (8-12)	10.01±2.88<br>9 (8-11)	F(1,5)=1.330, P=0.301	F(1,5)=0.205, P=0.669	F(1,5)=0.410, P=0.550
Elbow							
Flexion (°)	92.43±5.92<br>91 (89-94)	92.78±6.41<br>92 (89-98)	92.67±5.15<br>94 (91-96)	93.78±6.24<br>93 (90-99)	F(1,5)=0.445, P=0.534	F(1,5)=0.298, P=0.609	F(1,5)=0.445, P=0.534
Extension (°)	143.56±6.83<br>143 (137-148)	145.77±7.03<br>145 (139-153)	144.77±5.78<br>147 (143-150)	145.81±7.48<br>145 (139-153)	F(1,5)=1.440, P=0.284	F(1,5)=1.478, P=0.278	F(1,5)=1.059, P=0.351
ROM (°)	52.99±4.61<br>51 (49-53)	51.12±2.89<br>53 (51-56)	53.09±2.77<br>53 (51-55)	52.03±3.48<br>53 (51-54)	F(1,5)=1.453, P=0.282	F(1,5)=0.163, P=0.703	F(1,5)=5.228, P=0.071
Carpus							
Flexion (°)	101.42±7.86<br>101 (96-106)	103.31±8.03<br>104 (99-107)	105.76±7.61<br>103 (100-112)	104.87±6.69<br>104 (101-111)	F(1,5)=7.317, P=0.043	F(1,5)=0.068, P=0.804	F(1,5)=2.824, P=0.154
Extension (°)	183.74±2.98<br>184 (181-185)	183.22±3.27<br>183 (181-184)	183.72±4.82<br>183 (181-187)	182.46±4.00<br>182 (179-184)	F(1,5)=0.229, P=0.653	F(1,5)=1.189, P=0.325	F(1,5)=0.480, P=0.519
ROM (°)	82.31±7.31<br>84 (78-88)	79.91±8.30<br>82 (75-84)	77.95±8.04<br>80 (70-84)	77.59±8.73<br>79 (71-84)	F(1,5)=4.084, P=0.099	F(1,5)=1.129, P=0.337	F(1,5)=1.574, P=0.265
Fetlock							
Flexion (°)	148.21±10.37<br>147 (142-152)	148.49±10.22<br>148 (142-152)	150.78±9.50<br>151 (143-157)	151.15±8.75<br>152 (143-159)	F(1,5)=2.929, P=0.14	F(1,5)=0.695, P=0.442	F(1,5)=0.003, P=0.961
Extension (°)	236.05±2.96<br>236 (234-237)	235.47±4.27<br>236 (233-238)	234.27±6.75<br>237 (233-238)	234.73±5.66<br>235 (231-239)	F(1,5)=0463, P=0.506	F(1,5)=0.025, P=0.876	F(1,5)=0.002, P=0.958
ROM (°)	85.55±9.92<br>86 (77-92)	86.99±10.86<br>87 (82-97)	83.49±8.12<br>84 (77-90)	83.59±10.53<br>80 (77-94)	F(1,5)=3.747, P=0.111	F(1,5)=0.336, P=0.588	F(1,5)=0.316, P=0.598

Table S2. Mean ± standard deviation for Mean Pressure (kPa), Peak Pressure (kPa) and Mean Force (N) , on the cranial and caudal areas of the saddle, for standard (S) and anatomical girths (A) at 8 and 16kg tension (n=6 horses).

	S8	S16	A8	A16	Girth Type (S v A)	Girth Tension (8 v 16kg)	Girth Type*Tension
Mean Pressure (kPa)							
Cranial	16.73 ± 7.22	18.80 ± 7.71	15.13 ± 4.65	20.07 ± 5.35	F(1.5)= 0.003, P=0.962	F(1.5)= 10.81, P=0.022	F(1.5)= 2.38, P=0.184
Caudal	7.67 ± 2.53	8.33 ± 7.71	8.12 ± 2.58	8.50 ± 2.57	F(1.5)= 1.047, P=0.353	F(1.5)= 0.774, P=0.428	F(1.5)=401, P=0.554
Cranial vs Caudal	t(5) = 3.260, P=0.023	t(5) = 3.053, P=0.028	t(5) = 3.055, P=0.028	t(5) = 4.647, P=0.006			
Peak Pressure (kPa)							
Cranial	35.15 ± 10.54	37.43 ± 9.76	33.08 ± 2.28	38.80 ± 9.75	F(1.5)= 0.005, P=0.947	F(1.5)= 2.33, P=0.187	F(1.5)= 0.83, P=0.404
Caudal	30.77 ± 10.8	29.18 ± 7.35	32.83 ± 12.23	31.20 ± 9.64	F(1.5)= 1.76, P=0.242	F(1.5)= 0.886, P=0.390	F(1.5)= 0.002, P=0.964
Cranial vs Caudal	t(5) = 0.886, P=0.415	t(5) = 1.99, P=0.103	t(5) = 0.048, P=0.963	t(5) = 1.80, P=0.131			
Mean Force (N)							
Cranial	519.07 ± 233.29	575.33 ± 255.45	447.92 ± 210.22	581.92 ± 255.31	F(1.5)= 0.193, P=0.679	F(1.5)= 9.43, P=0.028	F(1.5)= 3.05, P=0.141
Caudal	146.12 ± 47.30	148.32 ± 43.67	152.23 ± 45.76	147.23 ± 55.29	F(1.5)= 0.090, P=0.776	F(1.5)= 0.031, P=0.868	F(1.5)= 0.298, P=0.609
Cranial vs Caudal	t(5) = 4.532, P=0.006	t(5) = 4.437, P=0.007	t(5) = 4.011, P=0.010	t(5) = 4.654, P=0.006			
